# Effect of Social Participation on the Physical Functioning and Depression of Empty-Nest Elderly in China: Evidence from the China Health and Retirement Longitudinal Survey (CHARLS)

**DOI:** 10.3390/ijerph17249438

**Published:** 2020-12-16

**Authors:** Dai Su, Zhifang Chen, Jingjing Chang, Guangwen Gong, Dandan Guo, Min Tan, Yunfan Zhang, Yanchen Liu, Xinlan Chen, Xinlin Chen, Yingchun Chen

**Affiliations:** 1 Department of Health Management, School of Medicine and Health Management, Tongji Medical College, Huazhong University of Science and Technology, Wuhan 430030, China; sudai@hust.edu.cn (D.S.); chenzhifang@hust.edu.cn (Z.C.); changjingjing@hust.edu.cn (J.C.); guangwengong@163.com (G.G.); guodandan@hust.edu.cn (D.G.); tanmin@hust.edu.cn (M.T.); zhangyunfan@hust.edu.cn (Y.Z.); ycliu97@hust.edu.cn (Y.L.); chenxinlan@hust.edu.cn (X.C.); chenxinlin@hust.edu.cn (X.C.); 2 Research Center for Rural Health Services, Hubei Province Key Research Institute of Humanities and Social Sciences, Wuhan 430030, China

**Keywords:** social participation, empty-nest elderly, health, China

## Abstract

This study aimed to explore the impact of social participation (SP) on physical functioning and depression among empty-nest elderly taking part in the fourth wave of the China Health and Retirement Longitudinal Survey (CHARLS, 2018). The instrumental variable (IV) method and propensity score matching (PSM) method were used to analyse the impact of SP. The two-stage regression results of the IV method showed that SP has a significant negative impact on the physical functioning scores of empty-nest elderly (β = −3.539, *p* < 0.001) and non-empty-nest elderly (β = −4.703, *p* < 0.001), and SP has a significant negative impact on the depression scores of empty-nest elderly (β = −2.404, *p* < 0.001) and non-empty-nest elderly (β = −1.957, *p* < 0.001). The results of the PSM method were basically consistent with the IV method. Compared with non-empty-nest elderly, SP had more positive effects on the depression of empty-nest elderly (Wald χ^2^ = 6.62, *p* = 0.010). Providing a friendly and supportive environment for the SP of empty-nest elderly was an important measure to promote healthy ageing. Targeted SP may be one of the greatest opportunities to improve the mental health of empty-nest elderly.

## 1. Introduction

Empty-nest elderly are defined as people over the age of 60 whose children are away from home and who do not have other relatives at home, are single, living with their spouses or seniors [1]. Population ageing plays a pivotal role in economic and political fields because of the changes in the population structure in China. According to the data of the sixth national census in 2010, the population of China over 60 years old is 178 million, which accounts for 13.26% of the total population [2]. In 2011, Chinese urban and rural empty-nest elderly households accounted for 49.7% and 38.3% of households, respectively [3]. The proportion of empty-nest elderly in China is expected to reach 90% by 2030, which means that more than 200 million will be empty-nest elderly [4].

With the increase of age, the physiological functions of empty-nest elderly continue to deteriorate and manifest as decreased mobility, cognitive decline and acute and chronic diseases. A study showed that empty-nest participants, in comparison with non-empty-nest participants, had lower levels of physical health (95% confidence interval (CI): (0.228–6.044)) [5]. Empty-nest elderly also face various psychological problems, including anxiety and depression. Another study found that the depression symptoms of empty-nest elderly were more common than non-empty-nest elderly (11.6% vs. 8.6%, *p*-value < 0.001) [6]. Another study showed that, compared with the non-empty-nest respondents in China, older adults living alone in China were more susceptible to depression symptoms (odds ratio (OR) = 1.194, 95%CI: (1.016–1.405)), whereas older adults living with a spouse in China only were not exposed to an elevated risk of being depressed (OR = 0.945, 95%CI: (0.847–1.055)) [7]. The empty-nest elderly are vulnerable to different disadvantageous situations and experience problems associated with old age, such as physical and psychological problems [8]. Therefore, finding an effective way to improve the physical functioning and depression of empty-nest elderly is essential.

Some studies have identified the factors influencing of the health of empty-nest elderly which include cognitive decline [9], social support [10], healthy behaviours and lifestyles [11,12], chronic conditions [13], physical disability [14], gender, age, educational level, marital status, type of residence, endowment insurance and health insurance [15,16,17,18].

While existing research on the general elderly population has also investigated the impact of social participation (SP) on the physical functioning and depression [19,20], few studies have investigated this variable in a population of empty-nest elderly. Specifically, few studies compare the health differences amongst empty-nest elderly who participate in social activities and those who do not. The differences between the two groups are therefore still unclear. This study aimed to determine whether participating in social activities affected the physical functioning and depression of empty-nest elderly, to compare health outcomes of those taking part in social activities and those who did not, to identify factors influencing participation in social activities, and compare the results of non-empty-nest elderly. This study hypothesised that SP has a greater impact on the physical functioning and depression of empty-nest elderly than non-empty-nest elderly and that empty-nest elderly who participate in social activities can obtain more social support.

## 2. Materials and Methods

### 2.1. Data Source

The data used in this study were collected from the wave 4 of the China Health and Retirement Longitudinal Survey (CHARLS). The CHARLS aimed to collect high-quality nationally representative data from Chinese residents aged 45 years and older to serve the needs of scientific research on the elderly. The baseline data collection for CHARLS was conducted in 2011 and included about 10,000 households and 17,500 individuals in 150 counties/districts and 450 villages/resident committees [21]. Wave 2 was conducted in 2013, wave 3 in 2015 and wave 4 in 2018. All data were made public one year after the end of data collection. A stratified (by per capita gross domestic product (GDP) of urban districts and rural counties) multi-stage (county/district-village/community-household) probability proportional to size random sampling strategy was adopted.

### 2.2. Variables Selection

#### 2.2.1. Outcome Variables

The outcome variable of this study was health status, which was measured by two indicators: physical functioning and depression.

Physical functioning was assessed by activities of daily living (ADL) and instrumental activities of daily living (IADL) [22,23,24,25]. ADL limitations were measured by any self-reported difficulty in any of the following ADL domains: bathing/showering, eating, dressing, getting into or out of bed, using the toilet or controlling urination and defecation. IADL limitations were measured by any self-reported difficulty in any of the following household activities: doing household chores, preparing hot meals, shopping for groceries, managing money and taking medications. Twenty questions were given, and the responses were rated as 0 (no difficulties), 1 (difficulties but still can be completed), 2 (difficulties, need help) or 3 (unable to complete). A higher score indicated a higher degree of physical dysfunction.

Depression was measured by the Center for Epidemiological Studies Depression Scale-10 (CES-D10) in the CHARLS questionnaire [26]. The CES-D10 comprised 10 questions about depression, and the answers included four options: (1) rarely, (2) some days (1–2 days per week), (3) occasionally (3–4 days per week) and (4) most of the time (5–7 days per week). Amongst the 10 questions, eight questions were negative statements, and two questions were positive statements. The answers were recorded as 0 (rarely) to 3 (most of the time) for the negative questions and were recorded as 3 (rarely) to 0 (most of the time) for the positive questions. The depression index was obtained from the sum of the scores of the 10 questions. A higher score indicated higher depression.

#### 2.2.2. Core Explanatory Variable

The core explanatory variable of the study was the SP of empty-nest elderly and non-empty-nest elderly. The CHARLS questionnaire asked respondents about their participation in 10 social activities in the past month. This question had 12 options: (1) interacted with friends, (2) played ma-jong, chess, or edcards or went to a community club, (3) provided help to family, friends or neighbours who do not live with you, (4) went to a sport, social or other kind of club, (5) took part in a community-related organisation, (6) did voluntary or charity work, (7) cared for a sick or disabled adult who does not live with you, (8) attended an educational or training course, (9) stock investment, (10) used the Internet, (11) other or (12) none of these. We chose the first 10 options as the type of social activities for empty-nest elderly and non-empty-nest elderly. If any of the options were selected, empty-nest elderly and non-empty-nest elderly were considered to have participated in social activities and were included in the SP group; otherwise, they were considered to not have participated in social activities and were included in the non-SP group.

#### 2.2.3. Control Variables

In addition to the variable SP, we also selected control variables that studies [8,27,28,29,30] have shown to affect the physical functioning and depression of empty-nest elderly and not empty-nest elderly, such as gender, age, education level, marital status, type of residence, pension insurance, social health insurance, still smoking, drinking, number of chronic illnesses and disability.

### 2.3. Statistical Analysis

#### 2.3.1. Processing of Missing Values

The k-nearest neighbour (k-NN) approach was used to impute missing data for some variables [31]. For a given empty-nest elderly or non-empty-nest elderly with missing values, the k-NN method identified the k-nearest empty-nest elderly or non-empty-nest elderly on the basis of Euclidean distance. Using these empty-nest elderly or non-empty-nest elderly, we then replaced missing values by using a majority vote for discrete variables and weighted means for continuous features. One advantage of using this method was that missing values in all features are imputed simultaneously without the need to treat features individually.

#### 2.3.2. Instrumental Variables Regression

In order to test the impact of SP on the physical functioning and depression of empty-nest elderly and non-empty-nest elderly, the following OLS model (model 1) was established:

(1) $$h_{i}\;=\;\alpha_{0}+\alpha_{1}SP_{i}+\delta X_{i}+\varepsilon_{i}$$

where i refers to the individuals in the survey, $h_{i}$ denotes the health status (physical functioning and depression) of the individual i, and $SP_{i}$ represents whether the individual i participated in social activities. $X_{i}$ refers to other control variables, $\varepsilon_{i}$ is the error term, $\alpha_{1}$ and δ are the parameters to be estimated and $\alpha_{1}$ represents the effect of SP on the physical functioning and depression.

We then turned to the instrumental variable method with two-stage regression estimation to address endogeneity and reverse causality problems. Affected by factors such as age and health, most of the daily contacts of the elderly are neighbours or other elderly in the village/community in addition to their spouses. Meanwhile, limited by geographical environment and traffic, their social participations were often concentrated in the village/community, which forms a social group with independent spaces and relatively stable relationships. Therefore, whether the elderly participate in social activities is highly susceptible to the influence of other elderly groups in the village/community, and the probability of the elderly participation in social activities in the village/community does not directly affect the physical functioning and depression of the elderly. Therefore, we used the probability of the elderly in the village/community participating in social activities as the IV of SP.

The basic idea of IV regression was to construct a two-stage estimation model by adding exogenous variables that are related to endogenous variables but not related to error terms to obtain a consistent estimate of the influence of endogenous variables on the explained variables. Besides, according to the empirical rule proposed by Staiger and Stock [32], in IV regression, if the F values of the first stage are greater than 10, the weak IV problem can be excluded. We built interaction terms in IV regression and conducted the Wald test to test the equality of coefficients of SP in two IV regression models. The model of IV (models 2 and 3) was constructed as follows:

(2) $$SP_{i}\;=\;\beta_{0}+\beta_{1}IV_{i}+\varphi X_{i}+\mu_{i}$$

(3) $$h_{i}\;=\;G\left(\alpha_{0}+\alpha_{1}\hat{SP_{i}}+\delta X_{i}+\gamma \hat{x_{ui}}\right)$$

In Equation (2), $IV_{i}$ represents the *IV* value of the individual i. In Equation (3), $\hat{SP_{i}}$ refers to the predicted value of *SP* in the first-stage model, and $\hat{x_{ui}}$ is the residual fitting value of the first-stage regression equation calculation.

#### 2.3.3. Propensity Score Matching

The outcomes between the SP group and comparable non-SP group were compared to estimate the effects of SP on the physical functioning and depression of empty-nest elderly and non-empty-nest elderly in China. Although the SP group can be easily selected from the CHARLS database, some characteristics of the non-SP group may be inconsistent with those of the SP group. Thus, we constructed an appropriate non-SP group by using PSM. The PSM method was widely used in estimating the effects of the intervention when randomised controlled trials are not feasible. PSM can identify individuals for the non-SP group with similar characteristics as the individuals in the SP group. We estimated the propensity score according to the abundant covariates in the logistic regression, that is, the probability of whether an individual enters the SP group, which refers to the probability of SP amongst empty-nest elderly and non-empty-nest elderly. The propensity score estimation model (model 4) was as follows:

(4) $$P\left(SP_{i}\;=\;1\right)\;=\;exp\left(\beta X_{i}\right)/\left[1+exp\left(\beta X_{i}\right)\right]$$

Kernel matching and radius matching were used in our main analysis to test the balancing property of each observed covariate and the overall balance between the SP group and non-SP group in the baseline and to verify the reduced sampling bias achieved through matching. We used a replacement kernel matching at the bandwidth of 0.06, and the kernel type was epan kernel. We defined $\varepsilon \;=\;0.25{\hat{\sigma }}_{pscore}$ in radius matching, where ${\hat{\sigma }}_{pscore}$ is the sample standard deviation of the propensity score. Propensity scores were used to match the counterfactual non-SP group samples of similar SP group and then we compared the differences in the dependent variables between the SP group and non-SP group to obtain the average treatment effect on the treated (ATT). The calculation formula of ATT (model 5) was as follows:

(5) $$\mathrm{ATT}=\;E\left[\left(h_{i1}|SP_{i}\;=\;1\right)\right]-E\left[\left(h_{i0}|SP_{i}\;=\;1\right)\right]$$

where $h_{i1}$ and $h_{i0}$ represent the physical functioning and depression of the individual i of SP and non-SP, respectively.

We then checked the balance of the means of covariates after matching by examining the standardised mean differences between the non-SP group and the SP group in the baseline (i.e., the baseline was also checked before and after matching). After matching, the bias should be ≤5% (or *p* > 0.1) to establish adequate matching. Then, we calculated the achieved percentage of the bias reduction and examined each covariate’s standardised bias percentage before and after matching. Finally, we determined whether kernel matching is appropriate based on the mean reduction bias and median reduction bias of the overall balance [33].

### 2.4. Ethical Statement

This study was approved by the Ethics Committee of the Tongji Medical College, Huazhong, University of Science and Technology (IORG No: IORG0003571).

## 3. Results

### 3.1. Selection of Study Participants

**Figure 1:**
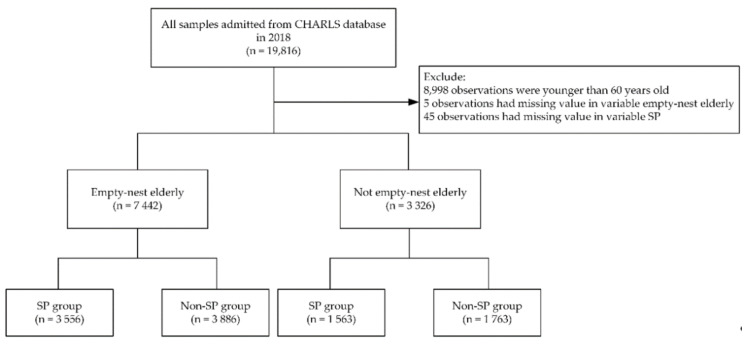
Selection of study participants. The n stands for the observations. CHARLS, China Health and Retirement Longitudinal Study; SP, social participation.

In this study, 19,816 observations were admitted from the 2018 CHARLS database. The inclusion criteria for this study were: all individuals included in the CHARLS 2018 database. The exclusion criteria were: (1) the participant was under 60 years old, (2) the variable empty-nest elderly of the participant was missing and (3) the variable SP of the participant was missing. After selection, 7442 empty-nest elderly and 3326 non-empty-nest elderly were separated into SP group and non-SP group, respectively (Figure 1).

### 3.2. Characteristics of the Study Sample

The descriptive statistics for all variables are shown in Table 1: 5287 (49.1%) participants were male, and their mean age was 69.3 ± 7.2 years, about 8065 (74.9%) participants were married and lived with their spouse, 194 (1.8%) participants were smokers and 5115 (47.5%) empty-nest elderly in the village/community participated in social activities. The average physical functioning score was 8.331 ± 10.473, and the average depression score was 8.875 ± 6.288. 

### 3.3. Effects of SP on the Physical Functioning and Depression of Empty-Nest Elderly Based on IV Regression

The regression results of SP on the physical functioning and depression of empty-nest elderly and non-empty-nest elderly estimated by ordinary least squares (OLS) and two-stage least squares (2SLS) are shown in Table 2.

According to the robust F-value of the first-stage regression of two models (robust F = 772.81, *p* < 0.001; robust F = 1089.68, *p* < 0.001), we determined that the regression does not have a weak IV, which means that the selected IV in this study are effective. The regression results of the first-stage regression showed that IV has a significant positive impact on the SP of empty-nest elderly (β = 0.930, *p* < 0.001) and non-empty-nest elderly (β = 0.961, *p* < 0.001).

In terms of physical functioning, the two-stage regression results showed that SP has a significant negative impact on the physical functioning scores of empty-nest elderly (β = −3.539, *p* < 0.001) and non-empty-nest elderly (β = −4.703, *p* < 0.001). This result indicated that more SP is related to lower physical functioning scores of empty-nest elderly and non-empty-nest elderly, that is, SP was more conducive to improving the physical functioning of the elderly. Moreover, compared with empty-nest elderly, SP had more positive effects on the physical function of non-empty-nest elderly (Wald χ^2^ = 17.30, *p* < 0.001).

In terms of depression, the results showed that SP has a significant negative impact on the depression scores of empty-nest elderly (β = −2.404, *p* < 0.001) and non-empty-nest elderly (β = −1.957, *p* < 0.001), which indicates that SP is more conducive to improving the depression of the elderly. Moreover, compared with non-empty-nest elderly, SP had more positive effects on the depression of empty-nest elderly (Wald χ^2^ = 6.62, *p* = 0.010).

Regardless of physical functioning or depression of empty-nest elderly, the absolute value of the estimated coefficient of 2SLS was greater than the coefficient estimated by OLS. This result indicated that SP has a more significant positive effect on the physical functioning and depression of empty-nest elderly after using IV estimation.

### 3.4. Effects of SP on the Physical Functioning and Depression of Empty-Nest Elderly Based on PSM Method

As shown in Table 3, the overall balancing properties can be verified by comparing the joint significance of all matching variables in the logit models before and after matching. In column 1, the pseudo R^2^ of the results after PSM (pseudo R^2^ = 0.000) in two groups by using kernel matching and radius matching were much lower for the matched sample than for the raw sample. In columns 2 and 3, the matching covariates were jointly significant before kernel and radius matching and became insignificant after kernel and radius matching in two groups. The results further indicated that kernel and radius matching have improved the overall balance after matching. Columns 4 and 5 consistently showed that the mean and median of the absolute standardised bias have been reduced substantially by kernel and radius matching.

The results of SP on the physical functioning and depression of two groups estimated by the PSM method are shown in Table 4. Similar to the results of IV regression, SP has a remarkable effect on the physical functioning and depression of empty-nest elderly.

Specifically, in terms of physical functioning, kernel matching results showed that empty-nest elderly (ATT = −2.622, *p*-value < 0.001) and non-empty-nest elderly (ATT = −3.238, *p*-value < 0.001) in the SP group had significantly lower physical functioning scores than those in the non-SP group, and the difference in radius matching (ATT = −2.586, *p*-value < 0.001; ATT = −3.216, *p*-value < 0.001) was similar to kernel matching. Similar to the results of IV regression, compared with empty-nest elderly, SP had more positive effects on the physical function of non-empty-nest elderly.

In terms of depression, the results of kernel matching (ATT = −1.091, *p* < 0.001) and radius matching (ATT = −1.049, *p* < 0.001) in the empty-nest elderly group showed that SP has a significant positive impact on the depression of empty-nest elderly. Moreover, in the empty-nest elderly group, the results of kernel matching (ATT = −0.926, *p* < 0.001) and radius matching (ATT = −0.907, *p* < 0.001) also showed that SP has a significant positive impact on the depression of empty-nest elderly. Similar to the results of IV regression, compared with non-empty-nest elderly, SP had more positive effects on the depression of empty-nest elderly.

## 4. Discussion

In this study, using the data from wave 4 of CHARLS, we applied IV regression and PSM methods to explore the impact of SP on the physical functioning and depression of empty-nest elderly. We found that SP can improve the physical functioning and depression of empty-nest elderly and different demographic characteristics, health behaviours, number of chronic diseases and disability, have a substantial impact on physical functioning and depression. In addition, compared with non-empty-nest elderly, SP has a more positive effect on the depression of empty-nest elderly. This study is also the first study to analyse the impact of SP on the physical functioning and depression of empty-nest elderly.

Our results showed that the positive impact of SP was remarkable for physical functioning as well as depression for empty-nest elderly and non-empty-nest elderly, which is consistent with the findings in the previous studies [34,35,36]. Some positive effects of SP on the physical functioning and depression of the elderly have been discussed in previous studies. Regarding physical functioning, the physical activities required for SP in the elderly enhance the motor functions required to maintain functional independence. In terms of depression, SP allows the elderly to develop social relationships, which leads to social support and recognition of social roles and may even play a role in alleviating depression. Since the association between SP and depression has been reported [37,38,39,40], and the level of depression can predict future physical dysfunction [41], this may be an important reason for explaining the relationship between SP and physical dysfunction.

We found that compared with non-empty-nest elderly, SP has a more positive effect on the depression of empty-nest elderly. SP is a key factor in healthy ageing [42], especially amongst empty-nest elderly. SP can produce positive health outcomes in depression [43,44,45]. The main mechanisms of the positive impact of SP in the elderly include: (1) sharing information in social networks, which helps the elderly make more scientific health and medical decisions to relieve symptoms of depression [46], (2) maintaining a positive mental status [47,48], which has a protective effect against a decline in physical functioning [49], and (3) maintaining more life purposes to make up for the loss of family members or friends who have died or moved [50].

We found remarkable differences in some background characteristics (gender, age, education level, marital status, pension insurance) and SP amongst empty-nest elderly in China. Studies have pointed out that as people of the same age and close relatives leave and die, ageing tends to increase the risk of social network shrinkage [51,52]. The emphasis on family needs over individual needs has been long embedded in Chinese culture [53]. Married people may need to care for their spouses or child at home, and this practice may reflect a lack of association with SP [54]. Empty-nest elderly who have pension insurance tend to have a guaranteed income and are less financially dependent on their children, and this encourages their SP [2]. Consistent with research findings in high-income countries [55], education level has a substantial positive impact on SP. A higher education level means a greater tolerance for social norms, an interest in social affairs and a stronger social support network for empty-nest elderly, which all encourage their involvement in SP [56].

## 5. Limitations

Several limitations of this study should be noted. Firstly, data from the wave 4 of CHARLS was gathered at the individual level; thus, this study lacks community-level variables, which brings some difficulties to the richness of instrumental variables. Secondly, the concept of empty-nest elderly came into being under the unique social and cultural background of China. Our study has not been compared with other countries. Thirdly, we did not select other control variables that may be related to SP and the health of empty-nest elderly, such as social network support. These variables should be further explored in future research.

## 6. Conclusions

Providing a friendly and supportive environment for the SP of empty-nest elderly is an important measure to promote healthy ageing. A further measure is to focus on meeting the specific physical needs of empty-nest elderly and provide personalised and differentiated services to empty-nest elderly with different background characteristics. Targeted SP may be one of the greatest opportunities to improve the mental health of empty-nest elderly. This intervention will also generate social benefits by increasing the community’s contribution to this group. Our findings will enable health service researchers and policy makers to understand how to intervene and improve the health of empty-nest elderly.

## Figures and Tables

**Table 1:** Sample characteristics, CHARLS 2018.

Variables	Total		Empty-Nest Elderly						Non-Empty-Nest Elderly					
	Mean	SD ^2^	Total		SP ^1^		Non-SP		Total		SP		Non-SP	
			Mean	SD	Mean	SD	Mean	SD	Mean	SD	Mean	SD	Mean	SD
**Dependent variables**														
Physical functioning	8.331	10.473	8.087	9.996	6.085	7.251	9.920	11.672	8.877	11.453	6.301	8.071	11.161	13.367
Depression	8.875	6.288	8.892	6.339	8.141	6.098	9.580	6.476	8.836	6.174	8.083	5.864	9.504	6.364
**Control variables**														
Gender	0.491	0.500	0.501	0.500	0.490	0.500	0.510	0.500	0.469	0.499	0.464	0.499	0.474	0.499
Age	69.252	7.151	69.398	6.931	68.777	6.687	69.967	7.101	68.925	7.612	68.062	7.008	69.690	8.034
Educational level	3.027	1.906	3.048	1.914	3.382	2.012	2.742	1.766	2.980	1.888	3.280	1.955	2.714	1.785
Marital status	0.749	0.433	0.778	0.416	0.772	0.419	0.783	0.412	0.686	0.464	0.701	0.458	0.672	0.470
Type of residence	0.266	0.442	0.260	0.439	0.328	0.470	0.197	0.398	0.281	0.450	0.340	0.474	0.229	0.420
Pension insurance	0.902	0.298	0.947	0.223	0.949	0.220	0.946	0.226	0.906	0.291	0.906	0.292	0.907	0.291
Social health insurance	0.945	0.227	0.900	0.301	0.916	0.278	0.885	0.319	0.941	0.236	0.946	0.227	0.937	0.243
Still smoking	0.018	0.134	0.018	0.132	0.021	0.143	0.015	0.120	0.020	0.139	0.020	0.142	0.019	0.138
Drinking	0.311	0.463	0.322	0.467	0.354	0.478	0.292	0.455	0.287	0.453	0.333	0.472	0.247	0.431
Number of chronic diseases	0.804	1.108	0.820	1.120	0.829	1.110	0.812	1.128	0.768	1.081	0.782	1.081	0.756	1.082
Disability	0.157	0.364	0.161	0.367	0.136	0.342	0.184	0.388	0.149	0.356	0.138	0.345	0.158	0.365
**Instrumental variable**														
Probability of elderly in the village/community participating in SP	0.475	0.160	0.478	0.174	0.541	0.168	0.420	0.159	0.470	0.223	0.575	0.204	0.376	0.195

^1^ SP, social participation; ^2^ SD, standard deviation.

**Table 2:** Estimating the effect of SP on the physical functioning and depression of empty-nest elderly using OLS and 2SLS regression.

Variables	Physical Functioning						Depression					
	Empty-Nest Elderly			Non-Empty-Nest Elderly			Empty-Nest Elderly			Non-Empty-Nest Elderly		
	OLS ^1^	2SLS ^2^ Regression		OLS	2SLS Regression		OLS	2SLS Regression		OLS	2SLS Regression	
		First Stage	Second Stage		First Stage	Second Stage		First Stage	Second Stage		First Stage	Second Stage
IV		0.930 ***			0.961 ***			0.930 ***			0.961 ***	
SP	−2.688 ***		−3.539 ***	−3.395 ***		−4.703 ***	−0.833 ***		−2.404 ***	−1.021 ***		−1.957 ***
Gender	−1.352 ***	−0.075 ***	−1.429 ***	−1.435 ***	−0.054 **	−1.552 ***	−1.365 ***	−0.075 ***	−1.507 ***	−1.721 ***	−0.054 **	−1.805 ***
Age	0.347 ***	−0.005 ***	0.343 ***	0.529 ***	−0.005 ***	0.523 ***	−0.021 *	−0.005 ***	−0.028 **	−0.003	−0.005 ***	−0.008
Educational level	−0.403 ***	0.026 ***	−0.371 ***	−0.460 ***	0.016 **	−0.417 ***	−0.383 ***	0.026 ***	−0.324 ***	−0.343 ***	0.016 **	−0.313 ***
Marital status	−0.019	−0.048 ***	−0.062	−0.788	−0.023	−0.798 *	−1.178 ***	−0.048 ***	−1.257 ***	−0.853 ***	−0.023	−0.860 ***
Type of residence	−0.859 **	−0.012	−0.769 **	−0.798 *	−0.016	−0.682	−1.888 ***	−0.012	−1.722 ***	−1.221 ***	−0.016	−1.137 ***
Pension insurance	−0.844 *	0.036 *	−0.810 *	−0.361	−0.022	−0.417	−0.190	0.036 *	−0.128	0.038	−0.022	−0.002
Social health insurance	0.232	0.038	0.253	−0.737	0.027	−0.699	0.244	0.038	0.282	0.184	0.027	0.211
Still smoking	−1.866 *	0.07 *	−1.821 *	−0.634	−0.015	−0.643	0.335	0.07 *	0.417	0.192	−0.015	0.185
Drinking	−2.455 ***	0.065 ***	−2.392 ***	−2.055 ***	0.076 ***	−1.919 ***	−0.825 ***	0.065 ***	−0.708 ***	−0.522 *	0.076 ***	−0.424
Number of chronic diseases	1.399 ***	0.001	1.402 ***	1.845 ***	0.004	1.855 ***	0.766 ***	0.001	0.773 ***	0.715 ***	0.004	0.722 ***
Disability	4.611 ***	−0.054 ***	4.554 ***	5.539 ***	0.002	5.519 ***	2.777 ***	−0.054 ***	2.672 ***	2.126 ***	0.002	2.112 ***
Constant	−13.102 ***	0.276 ***	−12.539 ***	−23.817 ***	0.301 **	−22.875 ***	13.133 ***	0.276 ***	14.174 ***	11.377 ***	0.301 **	12.051 ***
N	7442	7442	7442	3326	3326	3326	7442	7442	7442	3326	3326	3326
Wald χ^2^ for SP (*p*-value)	17.30 (<0.001)						6.62 (0.010)					
Robust F (*p*-value)	772.814 (<0.001)			1089.68 (<0.001)			772.814 (<0.001)			1089.68 (<0.001)		

Note: Standard errors in parentheses; * *p* < 0.05, ** *p* < 0.01, *** *p* < 0.001; ^1^ OLS, ordinary least squares; ^2^ 2SLS, two-stage least squares.

**Table 3:** Test of the overall balance from kernel and radius matching.

Group	Matching Methods	Sample	Pseudo R^2^	LR ^1^ χ^2^	*p* > χ^2^	Mean Bias	Median Bias
			(1)	(2)	(3)	(4)	(5)
Empty-nest elderly	kernel matching	Raw	0.041	422.86	<0.001	12.0	10.4
		Matched	0.000	4.38	0.957	1.6	1.2
	Radius matching	Raw	0.041	422.86	<0.001	12.0	10.4
		Matched	0.000	3.83	0.975	1.4	1.0
Non-empty-nest elderly	kernel matching	Raw	0.035	160.13	<0.001	10.6	5.5
		Matched	0.000	1.15	1.000	1.2	0.9
	Radius matching	Raw	0.035	160.13	<0.001	10.6	5.5
		Matched	0.000	0.48	1.000	0.7	6.6

Note: All results are computed using the Stata module of psmatch2; ^1^ LR, likelihood ratio.

**Table 4:** Estimating the effect of SP on the physical functioning and depression of empty-nest elderly using the PSM method.

Group	Dependent Variables	Matching Methods	ATT ^1^	S.E. ^2^	T-Stat	*p*-Value	95%CI ^3^	
							Lower	Upper
Empty-nest elderly	Physical functioning	kernel matching	−2.622	0.244	−10.95	<0.001	−3.091	−2.152
		Radius matching	−2.586	0.247	−12.22	<0.001	−3.001	−2.171
	Depression	kernel matching	−1.091	0.224	−8.58	<0.001	−1.341	0.842
		Radius matching	−1.049	0.227	−4.56	<0.001	−1.499	−0.598
Non-empty-nest elderly	Physical functioning	kernel matching	−3.238	0.406	−7.96	<0.001	−4.035	−2.441
		Radius matching	−3.216	0.414	−8.78	<0.001	−3.934	−2.498
	Depression	kernel matching	−0.926	0.156	−6.00	<0.001	−1.228	−0.624
		Radius matching	−0.907	0.158	−5.41	<0.001	−1.236	−0.579

Note: ^1^ ATT, average treatment effect among treated; ^2^ S.E., standard error; ^3^ CI, confidence interval; PSM, propensity score matching.
